# General practice and the New Zealand health reforms – lessons for Australia?

**DOI:** 10.1186/1743-8462-2-26

**Published:** 2005-11-02

**Authors:** Brian R McAvoy, Gregor D Coster

**Affiliations:** 1 Department of General Practice, School of Primary Health Care, Monash University 867 Centre Road, East Bentleigh, Victoria 3165, Australia; 2 Department of General Practice and Primary Health Care, University of Auckland Private Bag 92019, Auckland, New Zealand

## Abstract

New Zealand's health sector has undergone three significant restructures within 10 years. The most recent has involved a Primary Health Care Strategy, launched in 2001. Primary Health Organisations (PHOs), administered by 21 District Health Boards, are the local structures for implementing the Primary Health Care Strategy. Ninety-three percent of the New Zealand population is now enrolled within 79 PHOs, which pose a challenge to the well-established Independent Practitioner Associations (IPAs).

Although there was initial widespread support for the philosophy underlying the Primary Health Care Strategy, there are concerns amongst general practitioners (GPs) and their professional organisations relating to its implementation. These centre around 6 main issues:

1. Loss of autonomy

2. Inadequate management funding and support

3. Inconsistency and variations in contracting processes

4. Lack of publicity and advice around enrolment issues

5. Workforce and workload issues

6. Financial risks

On the other hand, many GPs are feeling positive regarding the opportunities for PHOs, particularly for being involved in the provision of a wider range of community health services. Australia has much to learn from New Zealand's latest health sector and primary health care reforms.

The key lessons concern:

• the need for a national primary health care strategy

• active engagement of general practitioners and their professional organisations

• recognition of implementation costs

• the need for infrastructural support, including information technology and quality systems

• robust management and governance arrangements

• issues related to critical mass and population/distance trade offs in service delivery models

## Review

### Preamble

The most recent New Zealand health reforms can be viewed from a wide range of perspectives, depending on whether you are a consumer, general practitioner, practice nurse, policy maker or health services manager. This review has been written from the general practice perspective by an Australian GP academic who has practised in both Australia and New Zealand and by a New Zealand GP academic. The paper is based on a review of policy and discussion documents, peer-reviewed publications, websites and discussions with New Zealand colleagues.

### Context

Over the past 15 years or so, across most developed nations of the world the combination of burgeoning medical technology, ageing populations and increased patient expectations has led to dramatic escalations in the costs of providing health and social care. In 2001 Australia spent US$2,504 per capita on health, 9.1% of its Gross Domestic Product (GDP). New Zealand, with 20% of Australia's population, spent US$ 1,710 per capita, 8% of its GDP [1]. Not surprisingly, reforms of health care and social welfare have been undertaken across Europe, North America, South Africa, Australia and New Zealand. Barbara Starfield's review of health care in 11 industrialised nations concluded that a primary care orientation is associated with lower costs of care, higher satisfaction of the population with its health services, better health levels and lower medication use [2]. The upsurge of interest in primary care at such high levels of policy-making in so many different countries is related to the recognition of its potential to limit the escalating costs of secondary and tertiary care.

## Background

New Zealand's health sector has been subject to continual change since the early 1990s, undergoing three significant restructures within 10 years. These recent developments in the funding and organisation of the New Zealand health sector have been reviewed by Ashton who notes that after a decade of turbulence the sector now appears to be more stable [3]. The 1991 Green and White Paper ushered in an era of market oriented reforms which assumed that a purchaser-provider split and competition between health care providers would result in more efficient delivery of health services and, implicitly, improved health outcomes [4]. The reforms were intended to:

• Increase choice and access for all New Zealanders in a health care system that was effective, fair and affordable

• Encourage efficiency, flexibility and innovation in health care delivery

• Increase accountability to purchasers

• Reduce hospital waiting times

• Enhance the working environment for health professionals.

Four Regional Health Authorities (RHAs) were established, and the hospital and community services previously provided by 14 Area Health Boards were reconfigured into 23 Crown Health Enterprises (CHEs). The CHEs were required to manage their resources in a business-like fashion with the objective of being 'as successful and efficient as comparable businesses that are not owned by the Crown' [5]. However, the anticipated benefits of this 'experiment with competition' were not delivered [6]. In 1996 a briefing to the incoming Minister of CHEs stated 'the health reforms have yet to yield the original expectations. By a range of measures the pace of performance seems, if anything, to have weakened since the advent of the reforms' [7]. The CHEs' experience raises questions about the degree to which business models such as quasi-markets can be applied to public health provision. There were reductions in general practice subsidies, erosion of practice nurse subsidies and many primary care services including maternity, well-child and sexual health services were fragmented [8]. On a more positive note there emerged Maori health providers, community health trusts and most significantly, Independent Practitioner Associations (IPAs). These are similar in many respects to Australian Divisions of general practice and UK primary care groups but are owned and controlled independently by GPs themselves.

Currently over 75% of New Zealand GPs are members of over 30 IPAs which vary in size from 7 to 340 GP members (mean 74), and there is now an IPA Council of New Zealand (IPAC). IPAs cover more than 800 community-based practices, attended by some 2,200 GPs and more than 2,000 practice nurses. Developments in contracting and alternative methods of funding and managing services were initially either resisted strongly or treated with caution by the majority of GPs. The main opposition was voiced by the GP Action Group and to a lesser extent, for a short period, by the then New Zealand General Practitioners Association and the New Zealand Medical Association (NZMA). Early successes in contracting, in budget holding for pharmaceutical and laboratory services and establishing new services, arising in part from budget holding savings, led to gradual and progressive recruitment of IPA membership [9]. In both New Zealand and Australia the 'public' provision of primary health care remains via market driven private practice. Fortunately the reforms did not have to deal with any market failures in the quasi-market arrangements.

In 1996 New Zealand's first proportionally elected National (conservative) led coalition government signalled a change of direction. A single purchaser, the Health Funding Authority, replaced the four RHAs, the CHEs became 'Hospitals and Health Services' and had their 'for profit' status removed. 'Cooperation' replaced 'competition' as the new political catch-cry [10]. During this time the IPAs consolidated, developing well-established infrastructures, including staff, information systems, clinical guidelines, peer discussion groups, and personalised feedback on clinical performance. They also began to develop expertise in budget holding for laboratory tests and pharmaceuticals, making savings to develop new and better services [11]. The IPAs made significant efforts to manage pharmaceutical and laboratory expenditure with the savings achieved providing significant funding for a variety of new service developments. The ability to use some of these savings was important for the development of the IPAs [12]. However, the acquisition of such funding from savings also led to ongoing conflict with the Health Funding Authority which became embroiled in bureaucratic controlling processes in order to endeavour to recoup some of that funding.

Further radical changes followed the election of the Labour led coalition in 1999. The main structural change was abolition of the Health Funding Authority and its replacement by 21 new District Health Boards (DHBs) commencing in 2001, comprising a majority of locally elected and a minority of ministerially appointed members, accountable to the Minister of Health [13]. This was intended to strengthen local democratic input to decisions. Funding was now to be allocated between DHBs according to a formula based on the local population weighted for relative health need. Coupled with these structural changes a series of national strategies have been developed to guide the system; these identify objectives and priorities for improving health and independence levels in the population, aim to reduce the 'health gap' between Maori and non-Maori, and specify how services should be delivered [10].

The New Zealand Health Strategy was published in 2000 [14]. This provided an overall framework for the heath sector, with the aim of directing health services at those areas that would provide the greatest benefit for the population, focusing in particular on tackling inequalities in health (see Table 1). Primary health care is one of five service delivery areas in the New Zealand Health Strategy (see Table 2), which identifies seven fundamental principles for the health sector (see Table 3), and out of a total of ten goals and 61 objectives, highlighted 13 population health objectives (see Table 4). Particular priorities included cancer, cardiovascular disease, diabetes and mental health. The New Zealand Health Strategy has set out the strategic direction for the development of health services in New Zealand, based on a model for improving health outcomes. The lesson for Australia is that Australia should have a national health strategy, including a national primary health policy. Many providers have argued this for years – to deaf Commonwealth ears.

**Table 1:** New Zealand Health Strategy Priority Objectives to Reduce Inequalities

• Ensure accessible and appropriate services for people from lower socio-economic groups
• Ensure accessible and appropriate services for Maori
• Ensure accessible and appropriate services for Pacific Peoples.

**Table 2:** New Zealand Health Strategy Service Delivery Priority Areas

• Public health
• Primary heath care
• Reducing waiting times for public hospital elective services
• Improving responsiveness of mental health services
• Accessible and appropriate services for people living in rural areas

**Table 3:** New Zealand Health Strategy Principles

• Acknowledging the special relationship between Maori and the Crown under the Treaty of Waitangi
• Good health and wellbeing for all New Zealanders throughout their lives
• An improvement in health status of those currently disadvantaged
• Collaborative health promotion and disease and injury prevention by all sectors
• Timely and equitable access for all New Zealanders to a comprehensive range of health and disability services, regardless of ability to pay.
• A high performing system in which people have confidence.
• Active involvement of consumers and communities at all levels.

**Table 4:** New Zealand Health Strategy Population Health Objectives

• Reduce smoking
• Improve nutrition
• Increase the level of physical activity
• Reduce the rates of suicide and suicide attempts
• Minimise harm caused by alcohol, illicit and other drug use to both individuals and the community
• Reduce the incidence and impact of cancer
• Reduce the incidence and impact of cardiovascular disease
• Reduce the incidence and impact of diabetes
• Improve oral health
• Reduce violence in interpersonal relationships, families, schools and communities
• Improve the health status of people with severe mental illness
• Ensure access to appropriate child health care services including well child and family health care, and immunisation.

A New Zealand Disability Strategy was also developed, with fifteen objectives [15]. A significant policy shift towards population-based approaches was signalled by the National Health Committee [16], based on a paper by Coster and Gribben who proposed new primary health organisations with a focus on population-based health outcomes [17]. The New Zealand Health Strategy and Disability Strategy both informed the Primary Health Care Strategy, published in February 2001 [18]. The latter is a key document and promised to achieve a new vision over five to ten years with the following:

• People will be part of local primary health care services that improve their health, keep them well, are easy to get to and coordinate their ongoing care

• Primary health care services will focus on better health for a population, and actively work to reduce health inequalities between different groups.

**Figure 1:**
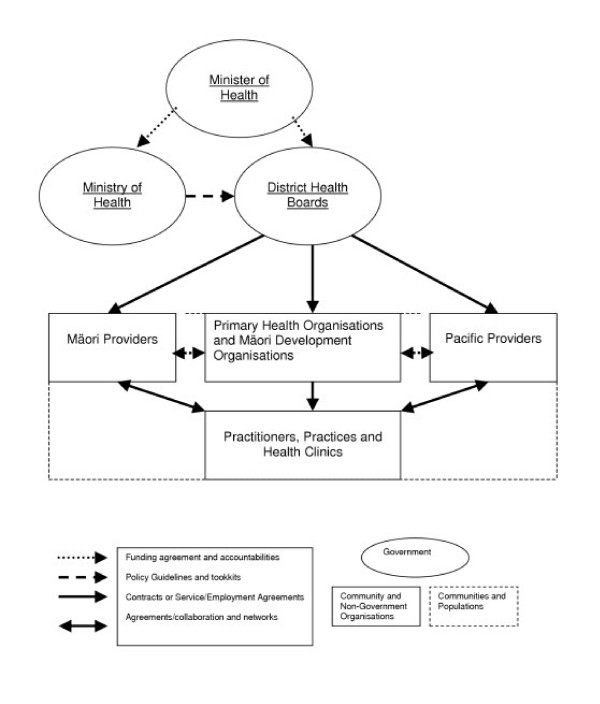
**The New Primary Health Care Sector**. This diagram reflects the sector as envisaged under this Strategy, however, as noted previously primary health care practitioners will be free to decide whether or not they join a Primary Health Organisation

Recent health policy developments in primary health care in New Zealand are redefining general practice to align with the 1978 Alma Ata Declaration [19]. The vision involves a new direction for primary health care with a greater emphasis on population health and the role of the community, health promotion and preventive care, the need to involve a range of professionals, and the advantage of funding based on population needs rather than fee for service. This reflects a desire by the New Zealand government to reduce health inequalities between different population groups, and protect and promote the health of its population. Central to the Primary Health Care Strategy are the new arrangements for primary health care, which are administered through the DHBs, supported by the Ministry of Health, which is the national policy advice, regulatory, funding and monitoring agency (see Figure 1). Primary Health Organisations (PHOs) are the local structures for implementing the Primary Health Care Strategy, and have the following features, set out in the Minimum Requirements released by the Health Minister in November 2001 [20]:

• PHOs will aim to improve and maintain the health of their populations and restore people's health when they are unwell. They will provide at least a minimum set of essential population-based and personal first-line general practice services

• PHOs will be required to work with those groups in their populations (for example, Maori, Pacific and lower income groups) that have poor health or are missing out on services to address their needs

• PHOs must demonstrate that they are working with other providers within their regions to ensure that services are coordinated around the needs of their enrolled populations

• PHOs will receive most of their funding through a population needs-based formula (capitation)

• PHOs will enrol people through primary providers using consistent standards and rules

• PHOs must demonstrate that their communities, iwi and consumers are involved in their governing processes and that the PHO is responsive to its community

• PHOs must demonstrate how all their providers and practitioners can influence the organisation's decision-making

• PHOs are to be not-for-profit bodies with full and open accountability for the use of public funds and the quality and effectiveness of services.

It is useful to clarify the relationships between IPAs and PHOs. IPAs, which were the original developments in organised general practice, have either been merged into or remain partially independent entities from PHOs. In general, and despite coming under the control of PHOs, IPAs have preserved the autonomy of general practice for both GPs and practice nurses. Whilst some loss of autonomy may be felt by rank-and-file GPs, this has been balanced to a certain extent by gains which organised general practice has made through the IPAs, and now especially through PHOs. The achievements have been significant in advancing the status and influence of general practice/primary health care. General practice, through PHOs, now has a much more important voice in its engagement within the health system, and particularly with DHBs, than was ever possible through individual general practice and general practice organisations.

The first two PHOs got underway in South Auckland in July 2002. To date 79 PHOs have been established with 3.72 million New Zealanders (93% of the population) enrolled. Cumming et al in a recent report note that there is a great variation between PHOs in terms of size, structure, age and context [21]. As a generalisation, there are two main types of PHOs (Table 5). Of the 77 PHOs established and studied as at April 2005, 38 were small with <20,000 enrolees; while these PHOs made up 49% of PHOs, they enrolled only 11% of the total enrolled population. Small PHOs tend to have difficulty in supplying management input within their organisation and meeting DHB requirements. Small PHOs are characterised by being made up of 76% access funded practices (see later); large PHOs are more commonly interim funded or mixed (72%) [21]. The issues surrounding critical mass are both interesting and vital. They also reflect the Australian problem of trading off population for distance in service organisation models – and implicit in this is how catastrophic risk can be managed across a population (e.g. a flu pandemic). If either country accepts a system design that does not provide a critical population under a population resource funding formula then we are setting up primary care to fail.

**Table 5:** Characteristics of PHOs (simplified)

**Small (< 20,000 enrolees)** Inadequate management resources	**Large (>20,000 enrolees)** Well resourced, efficiently managed
Access funded History – Previous NGO, capitated Low Investment in IT, premises Salaried doctors	Interim funded History – Previous IPA, fee-for-service Established IT, premises etc Doctors own practice

Low co-payments Full/increasing use of nurses Established community governance Maori and Pacific focus	Higher co-payments Use of nurses dependent on busy-ness Establishing community governance General population focus

The NZ Government has committed additional base funding of $NZ284 million for 2004/05, $NZ338 million for 2005/06 and NZ$425 million for 2006/07 to implement the Primary Health Care Strategy. The PHOs are funded under two formulae – Access and Interim. PHOs serving areas with people who have high health needs, i.e. Maori, Pacific Island people and those on low incomes, receive a higher level of funding, according to what is known as the Access Formula. Patients belonging to these PHOs are able to get free or very cheap visits to their GP. For example, a child under 6 years will pay NZ$14 to the practice; other age groups will pay between NZ$20–27 per consultation (normal total fee is approximately NZ$43–50). They also pay no more than $3 for a prescription. The remaining PHOs that are not on the Access Formula are funded according to the Interim Formula – so named because the Government would eventually like to see all PHOs on the Access Formula. Most patients belonging to PHOs on the Interim Formula will have to pay much the same as they do now to go to their GP. Subsides available with Community Services Cards and High Use Health Cards will still apply, but are intended to be phased out over time. Whilst historically the percentage of government funding of general practice has been low, it is now increasing but there still remain high and widely variable levels of co-payments [22].

Care Plus is a new service that was introduced to Interim PHOs in July 2004. It is aimed at people with significant chronic illness who need to visit a GP frequently. The service covers such conditions as diabetes, heart disease, mental health, terminal care and others. Care Plus provides an additional 10% capitation funding for these patients and 8.5% of PHO enrolled patients are eligible for Care Plus [23]. The key criterion is that the person is expected to need at least two hours of clinical contact time in the coming six months. All Care Plus patients will have a care plan developed for them, including quarterly reviews to check on health status, treatment, medications etc. The government introduced Care Plus around the time of the Interim Formula to assuage the concerns of the GPs who were not on the Access Formula and who felt that their high-needs patients were being unfairly disadvantaged. Care Plus aims to improve the management of chronic conditions, reduce health inequalities between population groups, improve teamwork within PHOs, and lower the cost for high-need patients [24]. An early evaluation suggests that this is a successful programme, with moderate levels of satisfaction among patients and the primary health care team. In the experience of the pilot practices, the time involved for patients and practitioners, patient apathy towards a more active role in their own care, and staffing, were the main barriers to implementation of the programme [25].

However, not all of the primary health care services will be supplied by all PHOs, and not all of the services will be subsidised by the government. From 1 October 2003 low cost healthcare for those under 18 years of age has been administered through the PHOs, and this was extended to cover all enrolled people aged 65 years and over from July 2004. Progressive introduction of the new funding means that those aged 18–24 were covered from 1 July 2005, and for 46–64 years are covered from 1 July 2006.

## Discussion

The Primary Health Care Strategy, launched in 2001, promised a new vision over five to ten years. Implementation of the Strategy is now well underway and the DHBs are in place and 79 PHOs have been established. Such radical changes will always be accompanied by teething problems, and it is still relatively early in the evolution of the changes to make absolute or definitive judgements. A formative evaluation on the development of DHBs has been published [26] and three early formative evaluations have been undertaken of the process of implementing PHO development [27-29]. The Royal New Zealand College of General Practitioners (RNZCGP) published an overview of the Primary Health Care Strategy implementation in April 2003 expressing concerns regarding the vulnerability of primary care early on in the reforms [30]. Also of relevance is the recently published report from NZMA indicating growing concerns regarding the New Zealand general practitioner workforce [31]. These issues are discussed further later. Typically, reforms and restructures happen with each parliament (in New Zealand 3 years), but a longer time frame was proposed in order to allow time for the primary care reforms to bed in properly. The recent re-election of the Labour-led Government for a third term in New Zealand should allow the reforms to be completed.

On a positive note the Primary Health Care Strategy has acknowledged the key role of general practice and primary health care in the health system. It is providing opportunities to:

• Address health inequalities

• Improve access to services

• Enhance population health, health promotion and preventative care

• Develop coordination and continuity of care

• Foster multidisciplinary care and collaboration.

There is goodwill present within the sector despite difficulties with the implementation processs, and a willingness to 'make it work' to achieve health gains for all [27]. Strong support has been expressed by PHOs for the philosophy of the Primary Health Care Strategy [21]. Although there was widespread support for the philosophy underlying the Primary Health Care Strategy both on its introduction and presently, there are concerns relating to its implementation from the RNZCGP, the NZMA and many general practitioners. These centre around six main issues:

1. Loss of autonomy

2. Inadequate management funding or support

3. Inconsistency and variations in contracting processes

4. Lack of publicity and advice around enrolment issues

5. Workforce and workload issues

6. Financial risks.

### 1. Loss of Autonomy

The majority of New Zealand GPs are self-employed and until recently only 30% of their income came from the public purse [30]. Despite being technically 'independent operators', running their own businesses and setting their own fees, they are significantly influenced by public policy. There is a long-standing tradition of autonomy, and strong suspicion of government moves to make GPs 'salaried servants'. The IPAs have been a great success story for general practice, bringing together GPs in an environment that embraces quality, education and accountability, coherent management and political strength [32]. IPAs have pioneered extensive development of information systems including merging and managing practice registers, analysing laboratory and pharmaceutical data, and providing personalised feedback to members. They have also formulated and monitored guidelines and pharmaceutical and laboratory services [12]. Nevertheless, about 20% of GPs have resisted the move to join IPAs. While some GPs have been persuaded for financial reasons to join, now at least 90% have joined either PHOs either directly or through IPAs, and 93% of the population is now enrolled with a PHO. There is a real risk of further fragmentation within general practice if the IPAs are not able to adapt to the transition to PHOs. Less than 25% of IPAs include members other than GPs and a few have community representation on their Boards [8], yet these are essential features of PHOs as set out in the Health Minister's minimum requirements.

GPs' fears of losing control of their destinies by becoming part of a PHO are perhaps best illustrated by considering an example. Partnership Health Canterbury Te Kei o Te Waka is the largest PHO in New Zealand with 350,000 enrolled individuals. Its governance body comprises nine individuals representing primary health care services and nine individuals representing broad consumer and community interests with an independent chair. These board members have been elected or selected by six electoral groups:

(on the provider side)

• General practice teams (GPs and practice nurses), as the contracted providers (5)

• Representatives of providers who are not general practice (4)

(on the consumer side)

• Maori (2)

• Pacific Island community (1)

• Territorial local authorities (2)

• Consumer and community representation (4)

This represents a very different balance of power compared to current IPA Boards. The IPAs are private organisations that may or may not hold government contracts. Although privately owned, the PHOs are more like instruments of government and are accountable as such to DHBs, which are crown owned entities. Consequently, there has been a considerable shift in the locus of control within primary care, one which the IPAs are presently resisting. More recently, GPs have expressed concerns regarding the potential for more controls, including restrictions on prescribing and laboratory testing. While these have not eventuated, there is significant mistrust of the Ministry of Health and Government [21]. One PHO (Middlemore) has folded because GPs and community representatives on the Board were at loggerheads on how the funding would be spent (Mike Lamont, personal communication). The other loss of autonomy comes in the form of the shifts in care, particularly to nursing, that is occurring within the GP practices. The advent of needs-based capitation means that patients can be seen and treated by any health professional including nurses where appropriate. Whilst this means that the nursing profession can feel that it's expertise can be properly recognised, GPs have felt threatened in some circumstances. Others have welcomed the change stating that it allows them to spend more time managing more complex consultations that require their skills.

### 2. Inadequate management funding and support

Concerns have been expressed that implementation funding is inadequate, particularly for management and health promotion [27]. Set up costs of PHOs have been high and establishment funding inadequate, especially for smaller PHOs. The report of the Referred Services Advisory Group to the Ministry of Health stated [33]:

'The group recognises that there are significant organisational and infrastructure costs involved in the functions required of PHOs. Much better information systems are required than many of those currently available, including at practice level. The group considers that the management payments currently proposed as part of the PHO funding are inadequate and should be increased. This will be particularly important as referred service savings will no longer be available to any PHO spending above its equitable level.' Management costs include those of governance, general management, planning control and coordination, performance monitoring and reporting, and referred services management.

If effective implementation of the Primary Health Care Strategy is to occur, these infrastructure needs and costs need to be much more clearly identified and addressed. Given the fragile position of many providers, it is also critical that the government funders ensure that payment mechanisms are efficient and timely. Late or omitted payments to providers do nothing to engender confidence or assist the viability of the organisations concerned.

Failure to recognise the cost of implementing reforms is also a common ongoing problem for Divisions of General Practice in Australia. The importance of proper management and governance has been illustrated in several Australian cases recently, including Divisions going under. Primary Care organisations do not have the luxury of going back to Treasury if things get tough. The need to develop robust governance arrangements is central to success.

These difficulties in New Zealand have been compounded by the failure to create an economic model of a PHO in order to understand the minimum size needed for viability, sustainability, and development. PHOs have enrolled populations varying in size from 2,000 to 350,000. In a recent review of the management costs for PHOs it was found that smaller PHOs (fewer than 20,000 enrolees) were not delivering on all required management services, primarily because they do not have the resources, including staff, to undertake all the requirements. Medium PHOs (between 21,000 and 75,000 enrolees) were better able to meet the requirements than small PHOs. So far the Ministry has not fully addressed the issue of management costs, and consequently significant numbers of PHOs are not meeting their targets for service delivery to patient populations [34]. Modelling and experience would suggest a critical mass of at least 100,000 as optimal (Jonathan Simon, personal communication). There are some lessons here for viability of some Australian Divisions with small populations in large areas of land. It is interesting to note that the UK Primary Care trusts have reached a similar conclusion – that populations of around 150,000 are needed for viability. This implies that there may be some trade offs in a pluralistic model in terms of performance and accountability expectations if small PHOs are deemed desirable.

The Ministry of Health has recently approved a Performance Management Programme by which PHOs will be assessed on their performance regarding 15 performance/quality indicators, including prescribing and laboratory use. This will provide additional funding to PHOs rewarding quality use of these services.

### 3. Inconsistency and variation in contracting processes

This was one of the main concerns identified in the Victoria University's Health Services Research Centre report [27].

• 'Inconsistency and variations in the contracting process was noted. Several different versions in the PHO contract have been signed. Not all PHOs had an agreed contract before going 'live'.

• 'Dissatisfaction was expressed over many of the processes involved with implementation of PHOs, especially with regard to funding and payment processes. Greater definition and clarity around the rules pertaining to qualification for funding was also felt to be required. Streamlining of payment processes, greater accuracy and appropriateness of reports, and timely payments, were believed to be necessary.'

Some of these difficulties have arisen due to a lack of clarity and consistency with regard to implementation of PHOs [35]. This is a reflection of the various degrees of enthusiasm and commitment to PHOs expressed by the 21 DHBs. The recent Victoria University Health Services Research Centre report has suggested that there are too many DHBs, leading to high transaction costs and duplication of effort [26]. There is evidence that the introduction of capitation has been supported with poor processes and business rules leading to gaming and inaccurate payments (Jonathan Simon, personal communication). More recently, DHBs are transitioning PHOs to the new PHO contract version 17 which amalgamates and standardises previous contracts.

### 4. Lack of publicity and advice around enrolment issues

Although the Primary Health Care Strategy promised 'a public education campaign to explain enrolment and promote its benefits for communities', there has been poor public awareness of PHOs and an associated lack of understanding by the general public as to the concept and implications of PHO enrolments. Duplicate enrolments, i.e. people enrolling in two or more PHOs, have been estimated to average 8.6% of the enrolled population [27], with a range of 1.6% to 13.6%. Tightening the rules of enrolment, particularly in regions which have more than one PHO in close proximity, has been considered important.

Further difficulties encountered have included large quarterly fluctuations in revenue as a result of mobile populations and rapid changes in numbers of enrolees in areas where new PHOs are being established [27].'Clawbacks' of funding between PHOs have created a new round of administration.

### 5. Workforce and workload issues

The New Zealand health reforms are unrolling against a backdrop of a substantial and growing shortage of GPs. In May 2004 the NZMA published *An Analysis of the New Zealand General Practitioners Workforce *[31], showing that the actual number of active GPs has decreased by 6.5% from 1997 to 2002 (and 8% over the last two of those years). Using the workforce definition 'GPs identifying general practice as their main type of work' the decrease is 13.4% from 1998 to 2002. Compared to Australia there are fewer GPs per 100,000 population. The report which collated information from many sources identified a number of key factors affecting the GP workforce:

• An ageing GP population

• Few new medical graduates choosing general practice as a career option

• The effects of increased numbers of women in the workforce

• The reliance on overseas-trained doctors

• GPs who are departing or intending to depart are not being replaced by incoming GPs

• Negative working conditions.

The workforce issues identified in this report are identical to those in Australia.

The report also concluded that 'if early action is not taken the problem will get progressively worse'. Compounding these concerns over workforce is a growing expectation that GPs will play an increasing role in chronic disease management, taking on responsibilities previously assumed by hospitals and secondary care providers. This requires additional time and skills, and is seen by some as a cost-shifting exercise – moving patients from hospital to community care. Finally, the additional administrative work (much of it non-reimbursed) associated with becoming part of a PHO is imposing increasing pressures on a shrinking, beleaguered workforce, with predictable effects on morale, especially for those GPs in small PHOs (<20,000). However, for those in large PHOs there is considerable centralised resource to provide management, administrative and clinical support.

### 6. Financial risks

Until recently only 30% of primary health care has been government subsidised, the balance being funded through private co-payments, health insurance and Accident Compensation payments [36]. In 2001, public sources of Vote Health in New Zealand were 76.7% (down from 82.4% in 1989) and private sources 23.3% (up from 17.6% in 1989) [37]. With the introduction of PHOs, levels and types of funding in general practice vary, particularly during this period of phased introduction of the new funding. This creates uncertainty of revenue for GPs during the establishment phase and high financial risk on an on-going basis [35]. This has recently been acknowledged by the Ministry of Health which has announced a 12-month 'funding floor' initiative for Access-funded PHOs and practices that have experienced financial hardship since becoming part of a PHO.

There is also a potentially destabilising effect associated with the establishment of low cost (i.e. more highly funded) PHOs located alongside practices that are being paid a lower level of subsidy. Although a majority of GPs have now joined PHOs, many have done so reluctantly. While joining a PHO can expose them to some financial risks, *not *joining is often not a viable option if other GPs in the district who are members of PHOs are able to reduce co-payments for their services [35]. However, since the new funding has been more fully introduced to the PHOs, GPs can expect a significant increase in their incomes. Many GPs have already benefited, reporting improved incomes and financial benefits from joining the PHOs. It is important to note that in most cases GPs are the unit of service provision in terms of payments from PHOs, even though practice revenues will also increase as a consequence of increased funding to the GPs who contribute to practice revenue. However, increasingly general practices (as companies, or other legal structure) are becoming the unit of service provision through a contract with the PHO. There are also indications that GPs, particularly in access funded practices, are now less worried and more supportive of the Strategy [21].

The RNZCGP has recently published a paper *The Public and Private Interface in New Zealand Primary Health Care *which states that 'in order to stabilise the primary care workforce, and achieve the intended outcomes of the Primary Health Strategy, it is essential that the interface between the private and public sector be more robustly and explicitly addressed. It is therefore critical that such a framework is developed as soon as possible and explicit models of engagement become an essential part of any government policy development, or contracting process with non-government providers' [36].

## Conclusion

The issues arising from the implementation of the Primary Health Care Strategy have been encapsulated well in the RNZCGP's recent report *The Public and Private Interface in New Zealand Primary Health Care *[36]. There is a broad range of issues arising from the implementation of the Primary Care Strategy. The following are identified for their relevance to the private/public interface.

1. DHBs started to contract with PHOs with considerable variation; the resulting outcome has been a shifting of hospital/DHB risk and responsibility on to primary care and in particular, general practice.

2. Minimal funding was allocated for infrastructure development, quality, information technology and governance capacity building.

3. Compliance and administrative demands ballooned for providers, with development of enrolment registers, information technology compatibility issues.

4. Government information technology and capacity were inadequate and underdeveloped for reporting and payment requirements – compromising provider viability.

5. Graduated funding introduction increased funding for those most at need, at risk populations became low-cost access PHOs while other PHOs took the interim funding formula. This created inequalities in boundaries, with instability of provider viability, and neighbouring practices being funded better resulting in patients leaving general practices to enrol with another down the road.

6. Some general practitioners are concerned about PHO governance requirements; where others in governance make strategic decisions directly affecting their (the GP) future. (The practitioner may be carrying personal financial risk such as capital investments, mortgages on homes to finance health services which are now affected by public governance requirements).

7. The gaps in knowledge and expertise of those in governance making strategic decisions affecting practices.

It is important to note that the majority of these issues are related to the administration, contracting and reporting of service provision, rather than the delivery of the service to the patient per se.

Evaluations undertaken so far using qualitative interviews with 12 PHOs and surveys of another 22, indicate that there was strong support for the philosophy that underlies PHOs, particularly the focus on populations, the increased collaboration across professional groups and the opportunity for improving service integration [27]. Not surprisingly, consumers are also strongly supportive of reduced co-payments.

The concluding paragraph of the evaluation of PHOs noted:

'However, there is still goodwill present within the sector, despite difficulties with the implementation process (hampered by delays in documentation regarding requirements); confusion and inconsistency in application of rules; and inadequate funding streams. There is also a willingness to 'make it work' for the longer term benefits to patients and the community, and to achieve overall health gains for the wider population. This is despite some concern that unless certain implementation difficulties are addressed, there is a danger that the restructuring of the primary care sector will not be viable in the long term' [27].

Now, two years later, the most recent evaluation of the implementation of the Strategy noted:

'In the four years since the government published the Primary Health Care Strategy much has been achieved and there is wide support for the goals of the Strategy. More than 90% of the population are enrolled in one of 77 Primary Health Organisations, an uptake considerably faster than originally anticipated. PHOs report that much of the set-up work has been completed and that effort can now be redirected towards substantive changes.

For many, better access has already been achieved through lower fees and PHOs report that they are better able to identify and meet the need of a known, enrolled, population. Community representation on PHO boards appears to be increasingly effective and many valuable initiatives are underway.

General medical practitioners, freed from the incentives of a fee-for-service subsidy, have noted a greater flexibility in how they use their time. Some have found in the PHO environment a welcome opportunity to cooperate with other practitioners and one went so far to say that the changes would rejuvenate general practice' [21].

Australia has much to learn from New Zealand's latest health sector and primary health care reforms. The key lessons concern:

• the need for a national primary health care strategy

• active engagement of general practitioners and their professional organisations

• recognition of implementation costs

• the need for infrastructural support, including information technology and quality systems

• robust management and governance arrangements

• issues related to critical mass and population/distance trade offs in service delivery models

## Competing interests

The author(s) declare that they have no competing interests.

## Authors' contributions

Brian McAvoy drafted the manuscript and Gregor Coster revised it, and added material from the latest evaluation of the New Zealand Primary Health Care Strategy.
